# Myokines as mediators of exercise-induced cognitive changes in older adults: protocol for a comprehensive living systematic review and meta-analysis

**DOI:** 10.3389/fnagi.2023.1213057

**Published:** 2023-07-13

**Authors:** Wouter A. J. Vints, Evrim Gökçe, Antoine Langeard, Iuliia Pavlova, Özge Selin Çevik, Mohammad Mosaferi Ziaaldini, Jasemin Todri, Orges Lena, Giorgos K. Sakkas, Suzanne Jak, Ioanna Zorba (Zormpa), Christina Karatzaferi, Oron Levin, Nerijus Masiulis, Yael Netz

**Affiliations:** ^1^Department of Health Promotion and Rehabilitation, Lithuanian Sports University, Kaunas, Lithuania; ^2^Department of Rehabilitation Medicine, Research School Care and Public Health Research Institute (CAPHRI), Maastricht University, Maastricht, Netherlands; ^3^Adelante Zorggroep Centre of Expertise in Rehabilitation and Audiology, Hoensbroek, Netherlands; ^4^Sports Rehabilitation Laboratory, Ankara City Hospital, Ankara, Türkiye; ^5^COMETE, INSERM, UNICAEN, Normandie Univ, Caen, France; ^6^Department of Theory and Methods of Physical Culture, Lviv State University of Physical Culture, Lviv, Ukraine; ^7^Department of Physiology, Mersin University, Mersin, Türkiye; ^8^Department of Exercise Physiology, Faculty of Sport Sciences, Ferdowsi University of Mashhad, Mashhad, Iran; ^9^Department of Physiotherapy, Universidad Catolica San Antonio (UCAM), Murcia, Spain; ^10^Lifestyle Medicine and Experimental Physiology and Myology Lab, Department of Physical Education and Sports Science, The Center of Research and Evaluation of Human Performance (CREHP), University of Thessaly, National and Kapodistrian University of Athens (TEFAA) Campus, Karyes, Greece; ^11^Research Institute of Child Development and Education, University of Amsterdam, Amsterdam, Netherlands; ^12^Information and Library Centre, University of Ioannina, Ioannina, Greece; ^13^Movement Control and Neuroplasticity Research Group, Group Biomedical Sciences, Catholic University of Leuven, Heverlee, Belgium; ^14^The Levinsky-Wingate Academic Center, Wingate Campus, Netanya, Israel

**Keywords:** biomarker, myokine, cognition, aged, exercise, physical activity, muscle, brain

## Abstract

**Background:**

The world’s population is aging, but life expectancy has risen more than healthy life expectancy (HALE). With respect to brain and cognition, the prevalence of neurodegenerative disorders increases with age, affecting health and quality of life, and imposing significant healthcare costs. Although the effects of physical exercise on cognition in advanced age have been widely explored, in-depth fundamental knowledge of the underlying mechanisms of the exercise-induced cognitive improvements is lacking. Recent research suggests that myokines, factors released into the blood circulation by contracting skeletal muscle, may play a role in mediating the beneficial effect of exercise on cognition. Our goal in this ongoing (living) review is to continuously map the rapidly accumulating knowledge on pathways between acute or chronic exercise-induced myokines and cognitive domains enhanced by exercise.

**Method:**

Randomized controlled studies will be systematically collected at baseline and every 6 months for at least 5 years. Literature search will be performed online in PubMed, EMBASE, PsycINFO, Web of Science, SportDiscus, LILACS, IBECS, CINAHL, SCOPUS, ICTRP, and ClinicalTrials.gov. Risk of bias will be assessed using the Revised Cochrane Risk of Bias tool (ROB 2). A random effects meta-analysis with mediation analysis using meta-analytic structural equation modeling (MASEM) will be performed. The primary research question is to what extent exercise-induced myokines serve as mediators of cognitive function. Secondarily, the pooled effect size of specific exercise characteristics (e.g., mode of exercise) or specific older adults’ populations (e.g., cognitively impaired) on the relationship between exercise, myokines, and cognition will be assessed. The review protocol was registered in PROSPERO (CRD42023416996).

**Discussion:**

Understanding the triad relationship between exercise, myokines and cognition will expand the knowledge on multiple integrated network systems communicating between skeletal muscles and other organs such as the brain, thus mediating the beneficial effects of exercise on health and performance. It may also have practical implications, e.g., if a certain myokine is found to be a mediator between exercise and cognition, the optimal exercise characteristics for inducing this myokine can be prescribed. The living review is expected to improve our state of knowledge and refine exercise regimes for enhancing cognitive functioning in diverse older adults’ populations.

**Registration:**

Systematic review and meta-analysis protocol was registered with the International Prospective Register of Systematic Reviews (PROSPERO) on the 24th of April 2023 (registration number CRD42023416996).

## 1. Background

### 1.1. Rationale

Since 1800, life expectancy has increased threefold, rising from 28.5 to 73.3 years in 2019, with a further increase of 6.6 years in the last 20 years alone, rising from 66.8 years in 2000 to 73.4 years in 2019. However, healthy life expectancy (HALE), which measures the average number of years a person can expect to live in good health, has only increased by 5.4 years in the last two decades, from 58.3 years in 2000 to 63.7 years in 2019 (World Health Organization [WHO], n.d.). With respect to the brain and cognition, advancing age is the main risk factor for neurodegeneration and cognitive decline (Hou et al., 2019). However, in the last 20 years, dementia prevalence has increased faster than one could explain from the increased proportion of older adults in our society alone (Mattiuzzi and Lippi, 2020; Davis et al., 2022), and is prospected almost to triple from 2019 to 2050 (Nichols et al., 2022). This highlights that the issue of a rising number of older adults with cognitive impairment extends beyond the problem of an aging society and suggests an increasing role of other risk factors related to dementia. For example, the prevalence of sedentary behavior, a known risk factor of cognitive decline and dementia has also increased in the last 20 years (Livingston et al., 2020; López-Valenciano et al., 2020). Sedentary behavior is characterized by an energy expenditure ≤1.5 metabolic equivalents (METs), while in a sitting, reclining or lying posture, and it is distinctly different from physical inactivity defined as insufficient physical activity level to meet physical activity recommendations (Tremblay et al., 2017). It has been shown that older adults spend on average 80% of their time in a seated posture and with 67% being sedentary for more than 8.5 h per day (Chastin et al., 2021). The repercussions of age-related cognitive decline are far-reaching, affecting not only the individual, but also their family and society as a whole. Cognitive decline has a substantial impact on an individual’s quality of life (Bárrios et al., 2013). Moreover, caring for cognitively impaired individuals can be particularly stressful for family caregivers and health care professionals (Kasper, 1990). Furthermore, cognitive decline is the largest contributor of societal, health care system and personal costs related to limitations in functional independence in community dwelling older adults (Falck et al., 2022).

The effects of physical activity on cognition and brain health among the rapidly growing older population have been widely researched and the benefits are well-documented (e.g., Zhao et al., 2022). The literature usually distinguishes between physical activity and exercise, whereby physical activity refers to any bodily movement that is produced by the skeletal muscles and that increases energy expenditure compared to resting. The term exercise, a subcategory of physical activity, refers to planned, structured, and repetitive physical activity, and is more specifically designed to improve certain fitness components, such as cardiorespiratory fitness, flexibility, balance, coordination, strength, and/or power (Bangsbo et al., 2019). Importantly, the effect of exercise on cognition has been reported in healthy older adults (e.g., see review Falck et al., 2019), older adults with mild cognitive impairment (Tam et al., 2022), and older adults with dementia (Zhang et al., 2022). Specifically, physical activity has been shown to have positive effects on executive functions (Chen et al., 2020), information processing speed (Lin et al., 2021), cognitive inhibition (Boucard et al., 2012), cognitive flexibility (Lerche et al., 2018), memory (Wu et al., 2021), and visuospatial ability (Nemoto et al., 2020). Even a single session of exercise (i.e., acute exercise) can transiently improve performance in various cognitive domains (Levin et al., 2021; Griebler et al., 2022). Studies investigating different modes (aerobic, resistance, balance, exergames, coordination, etc.) or doses of exercise (intensities, durations, or number of sessions per week) generally conclude that all are effective (e.g., Borde et al., 2015; Levin et al., 2017; Netz, 2019; Gallardo-Gómez et al., 2022).

Neuroimaging techniques developed along the years employed for examining the relationship between brain health, cognition and exercise have significantly expanded the understanding of the effect of chronic exercise [e.g., MRI –Erickson et al. (2011); fMRI –Chen et al. (2019); fNIRS –Eggenberger et al. (2016); EEG –Schättin et al. (2016); PET –Jonasson et al. (2019); 1H-MRS –Sheoran et al. (2023)], as well as acute exercise on brain health and cognition (e.g., Hsieh et al., 2018; Callow et al., 2021).

However, in-depth knowledge of the underlying mechanisms of the exercise-induced cognitive improvements is still incomplete (Pedersen, 2019; Liang et al., 2022; Vints et al., 2022b). Furthermore, the knowledge on the effect of physical activity on multiple organ systems is limited (Hawley et al., 2014; Sanford et al., 2020). The increased metabolic activity produced by contracting skeletal muscles elicits a challenge to the whole-body homeostasis, generating a distress in numerous cells, tissues, and organs. To meet this challenge, multiple integrated networks are operated, communicating between the muscles and the other organs, thus mediating the beneficial effects of exercise on health and performance (Hawley et al., 2014). The dynamics of these multiple complex communication pathways is not yet understood. Interestingly, a recently established comprehensive USA National Institute of Health (NIH) project, aiming to map the molecular transducers involved in response to both acute and chronic exercise, has declared that “Exercise provides a robust physiological stimulus that evokes cross-talk among multiple tissues that when repeated regularly improves physiological capacity, benefits numerous organ systems, and decreases the risk for premature mortality” (Sanford et al., 2020).

In the last few years, research on the effect of exercise on cognition is focusing on the cross-talk between bioactive substances released by physical exercise (called “exerkines”) and the brain (e.g., Liang et al., 2022; Vints et al., 2022b). Exerkines enable beneficial crosstalk between various systems, organs, and tissues, including regulation of metabolism and inflammatory responses, exertion of protective effects within the central nervous system, and enhancement of cognitive function (Liang et al., 2022).

Skeletal muscle mass comprises one of the most prevalent tissues in the human body, accounting for approximately 40% of the total body weight (Frontera and Ochala, 2015). Besides regulating various homeostatic processes, recent findings indicate that muscle-derived exerkines play an important role in mediating changes in cognitive function in response to physical exercise (Scisciola et al., 2021; Oudbier et al., 2022; Vints et al., 2022b). Muscle-derived signaling molecules can target the central nervous system (Pedersen, 2019; Onyango et al., 2021), eliciting responses from neurons and glial cells. In 2003, the first muscle-derived exerkine, the cytokine interleukin-6 (IL-6), was discovered and named “myokine” (Pedersen et al., 2003). Since then, more muscle-derived proteins were found, identifying skeletal muscle as an endocrine organ, and broadening the term myokine to “all cytokines and other peptides produced, expressed, and released by muscle fibers that exert paracrine, autocrine, or endocrine effects” (Pedersen and Febbraio, 2008). Also lactate, who has long be categorized as a myometabolite instead of a myokine, is now referred to as a myokine due to its endocrine effects (Brooks et al., 2023). In addition, the enzyme kynurenine aminotransferase, which is expressed by skeletal muscle, and kynurenine-derived metabolites have been called myokines, although kynurenine itself is produced by the liver (Rai and Demontis, 2022). By now, research has identified over 600 myokines (Görgens et al., 2015). However, their specific bioactivity remains largely undescribed and poorly understood (Lee and Jun, 2019) and most of the myokines likely exert paracrine, not endocrine effects (Weigert et al., 2014). However, some myokines cross the blood-brain barrier and signal directly to brain cells, while others activate specific signaling cascades from outside the brain exerting an indirect effect on the brain. Whether direct or indirect, myokines have been shown to facilitate the cross-talk between muscle and brain, indicating that they may mediate a muscle-brain endocrine loop (Chen et al., 2021). Hence, many studies have investigated the function of myokines secreted following acute or chronic exercise to better understand how exercise affects cognition. Overall, most work has been done with aerobic type of exercise, while other exercise modes such as resistance exercise, mind-body exercise or multimodal exercise also induce the release of myokines (Parada-Sánchez et al., 2022; Solianik et al., 2022; Vints et al., 2022b). Moreover, clinical evidence indicates that older adults with sarcopenia [a loss of muscle strength and muscle mass and/or physical performance (Cruz-Jentoft et al., 2019)] suffer cognitive impairment (Cipolli et al., 2019; Ramoo et al., 2022) and this is suggested to be linked to myokine levels (Scisciola et al., 2021; Oudbier et al., 2022). Therefore, understanding the association between muscle-derived signaling factors and cognition may be a promising avenue for interventions aimed at promoting healthy aging.

### 1.2. What myokines are known to impact cognitive function?

Over 1,125 putative myokines have been described in human secretome or transcriptome studies (see section “2.1.5. Outcomes”; for a full list, see Supplementary Appendix B) and more myokines may be discovered in the next years. Several of these myokines have been identified as potential mediators of exercise-induced cognitive function changes. While some of these myokines are commonly examined in human exercise-cognition studies, others are promising candidates that have only been studied in animal or *in vitro* studies (see reviews of Piccirillo, 2019; Rai and Demontis, 2022; Vints et al., 2022b).

Here, we introduce some of the myokines that have most frequently been linked to cognition, including: brain-derived neurotrophic factor (BDNF), fibronectin type III domain-containing protein 5 (FNDC5)/irisin, cathepsin B (CTSB), insulin-like growth factor-1 (IGF1), IL-6, and L-lactate (Scisciola et al., 2021). For a review paper, which describes the molecular signaling pathways related to neuroplastic processes of these myokines and other exerkines, see Vints et al. (2022b). Within the living meta-analysis, we will assess if there exists evidence for a role of any of the more than 1,125 putative myokines in the exercise-cognition relationship.

The neurotrophin known as BDNF is reported to have a predominant role in neuronal growth, repair, and regeneration (Huang and Reichardt, 2001; Hultman et al., 2014; McGregor and English, 2019). There is reliable evidence that both acute and regular exercise significantly affect BDNF levels in diseased and healthy populations (Szuhany et al., 2015). Several studies have reported that a possible mechanism underlying improved cognitive function following exercise may partly be related to muscle-derived BDNF (Huang et al., 2021). Considering that higher BDNF levels positively affect hippocampal functioning and verbal/episodic/spatial memory (Mizuno et al., 2000; Vaynman et al., 2004; Grassi-Oliveira et al., 2008; Canivet et al., 2015), exercise has been seen as a potential candidate for increasing BDNF signaling to improve cognition. Following exercise, BDNF levels have been shown to increase both in the brain (Seifert et al., 2010), and in muscle (Matthews et al., 2009). Both may lead to increased BDNF levels in the peripheral circulation (Máderová et al., 2019; Huang et al., 2021), as BDNF is reported to cross the blood-brain barrier bi-directionally (Pan et al., 1998). Hence, further research is needed to understand the source of BDNF associated with improved cognition. Indeed, the effect may arise from other myokines that induce brain-derived BDNF following exercise, such as irisin and CTSB.

Irisin is produced from proteolytic cleavage of PGC-1α transmembrane precursor FNDC5 and upregulated following exercise in the skeletal muscle and hippocampal neurons. FNDC5/irisin crosses the blood-brain barrier (Wrann et al., 2013; Islam et al., 2021) and induces the expression of BDNF centrally, thereby participating in improved cognition (Boström et al., 2012). Moreover, irisin deficiency inhibits cognitive performance in exercise and aging (Wrann et al., 2013).

Cathepsin B was demonstrated to increase in the blood circulation after exercise, cross the blood–brain barrier and induce the hippocampal expression of BDNF, accompanied by spatial memory improvement (Moon et al., 2016). Although few studies on the relationship between exercise, CTSB, and cognitive performance have yielded controversial results, increased CTSB and improved cognitive performance have been reported following exercise (Gaitán et al., 2021; Micielska et al., 2021), implicating CTSB as a mediator of the cognitive effects of exercise.

Insulin-like growth factor-1, similar to BDNF, is a multifunctional peptide associated with neural development, neurogenesis, synaptogenesis (D’Ercole et al., 2002; Cheng et al., 2003; Guan et al., 2003), and has neuroprotective effects following nerve injury. Carro et al. (2000) demonstrated that IGF-1 crosses the blood-brain barrier, in turn, regulates hippocampal BDNF expression (see also Vints et al., 2022b). In humans, physical activity causes a rapid increase in peripheral IGF-1 levels (Berg and Bang, 2004; Gulick et al., 2020). Additionally, chronic exercise has been associated with increased peripheral IGF-1 levels modulated by age and gender effects (Jiang et al., 2020). Studies focusing on the relationship between exercise, IGF-1 and cognition have demonstrated that exercise improves circulating IGF-1 and cognition, depending on exercise type, duration, and gender (Stein et al., 2018).

Interleukin-6 increases dramatically in response to exercise (Pedersen and Fischer, 2007). In muscle cells, the IL-6 gene remains silent in rest but is activated by muscle contraction (Pedersen et al., 2003). IL-6 can cross the blood-brain barrier (Banks et al., 1994), indicating that muscle-derived IL-6 may affect the brain. IL-6 levels were related to neurometabolic changes reflecting neurodegenerative processes (Vints et al., 2022a). Acute exercise increases peripheral IL-6 (Kuhne et al., 2023), whereas chronic exercise decreases IL-6 levels and improves cognition (Alghadir et al., 2021; Qi et al., 2021). However, due to IL-6 activating pro-inflammatory and anti-inflammatory processes depending on the condition, further research is needed to improve understanding of the exercise-induced neuroinflammation pathway.

L-lactate is the end product of glycolysis, released from many tissues, including skeletal muscle, acting as a signaling molecule in the brain. It plays a role in learning and memory (Suzuki et al., 2011), adult hippocampal neurogenesis (Lev-Vachnish et al., 2019), and modifies neuron excitability (Sada et al., 2015). Following exercise, lactate levels increase in the peripheral circulation and in the hippocampus (Ide et al., 2000). El Hayek et al. (2019) demonstrated that exercise-induced peripheral lactate crosses the blood-brain barrier, and promotes hippocampal BDNF expression, thereby improving learning and memory. Furthermore, lactate can serve as a precursor for glutamate, an excitatory neurotransmitter which is also critical for learning processes (Maddock et al., 2016).

### 1.3. Objectives

Our goal in this review is to map the possible pathways between exercise-induced myokines and specific cognitive domains enhanced by exercise. By doing so, we aim to contribute to the understanding of the molecular transducers involved in response to both acute and chronic exercise. Specifically, our primary research question is whether exercise-induced myokines serve as mediators of cognitive function in older adults.

Additional sub-questions are:

1. What specific cognitive components are associated with exercise-induced changes in myokine levels?
2. What specific myokines are associated with exercise-induced changes in cognition?
3. To what extent do exercise characteristics (i.e., type of exercise, intensity of exercise intervention duration, frequency of exercise bouts per week, and exercise session length) moderate the effect of exercise on myokine levels in exercise-cognition studies?
4. To what extent do participant characteristics [i.e., cognitive health, age, sex, body mass index (BMI), educational level, physical fitness level, and comorbidities] moderate the effect of exercise on myokine levels in exercise-cognition studies?
5. Which of the studied myokines can be considered as mediators of cognition and to what extent do different myokines affect different cognitive components?

Our search in PubMed (without applying filters, see Supplementary Appendix A) revealed that this topic is relatively new, but rapidly growing. Before 2002, less than 100 articles were published per year on this topic, whereas since 2020, more than 600 articles are being published annually. Considering the rapid pace at which new evidence is being generated, we intend to conduct this review as a living review.

A living review is continually updated in a predetermined period, incorporating relevant new evidence as it becomes available, and is particularly important in fields where research evidence is emerging rapidly, current evidence is uncertain, and new research may change policy or practice decisions (Elliott et al., 2017). This new evidence synthesis method is being trialed as one of the outcome products of PhysAgeNet, a European Cooperation in Science and Technology (COST) network.^1^ PhysAgeNet aims to develop and sustain an international knowledge community about physical exercise at an older age. We plan to update our living systematic search every 6 months, for a minimal period of 5 years.

## 2. Methods and analysis

We plan to carry out the living systematic review with meta-analysis in the second half of the year 2023. The methods of this systematic review with meta-analysis were registered in the International Prospective Register of Systematic Reviews database (PROSPERO) on the 24th of April 2023 (registration number CRD42023416996).

Prior to starting this project, we searched PubMed, PROSPERO, and Cochrane Central Register of Controlled Trials (CENTRAL) to confirm that no systematic reviews or meta-analyses with the same research questions had recently been published or were currently being conducted. The protocol of this systematic review and meta-analysis is in accordance with the Preferred Reporting Items for Systematic Review and Meta-analysis Protocols (PRISMA-P) guidelines (Shamseer et al., 2015).

### 2.1. Eligibility criteria

Studies will be selected according to the criteria outlined below.

#### 2.1.1. Study designs

To ensure the highest quality of evidence, we will only include randomized controlled trials and exclude non-randomized controlled trials, cross-over studies, or studies that lack pre-to-post exercise comparisons such as case-control studies, or those that do not include a control group, such as longitudinal studies, case reports, or case series. Additionally, we will also exclude guidelines and expert opinions.

We will conduct a search for review papers and gray literature, which include article synopses, unpublished studies such as Ph.D. theses or master theses, conference abstracts, and trial registrations. We will evaluate these sources to determine whether they lead to additional randomized controlled trials that meet our inclusion criteria. In case gray literature is retrieved as potentially eligible, we will contact the authors of this literature to request any associated peer-reviewed publications or published data.

#### 2.1.2. Participants

Studies with participants aged 50 years or older with a mean age of 60 years or above will be included, regardless of their health status. Participants may have existing medical conditions or be in good health. Animal studies will be excluded.

#### 2.1.3. Interventions

We will include interventions described as a single bout (acute) or multiple bouts (chronic) of voluntary exercise. We define voluntary exercise as a planned or structured bodily movement done to improve or maintain one or more components of physical fitness [based on the definition of the American College of Sports Medicine, 2001 (Jakicic et al., 2001)]. Examples are: cardiorespiratory endurance exercise, high-intensity interval training, resistance exercise, mind-body exercises, balance training, multicomponent exercise, or other specific exercises that do not fit in any of these categories.

We will exclude exercise interventions that do not include a voluntary component (e.g., electrical stimulation and whole-body vibration) or involve solely passive muscle fiber movement (e.g., stretching and manipulations). Additionally, we will exclude exercise interventions that involve a component of cognitive training (e.g., dual task training) or the addition of a nutritional intervention. Finally, we will exclude routine daily activities or occupational tasks that do not meet the definition of exercise based on the American College of Sports Medicine (2001) (Jakicic et al., 2001).

#### 2.1.4. Comparators

Only studies with a control group will be included. The following control groups are eligible for inclusion: passive controls (e.g., waitlist), treatment as usual, active non-exercise controls (stretching, puzzle, computer games not targeting specific cognitive functions, and non-exercise recreational activities), non-active non-therapeutic activities (e.g., health education and non-exercise recreational activities). We will exclude studies that solely compare two exercise interventions, including all exercise interventions listed as possibly included interventions in section “2.1.3. Interventions,” without another control group. Studies that use cognitive training or dual tasking interventions as a control condition will also be excluded.

#### 2.1.5. Outcomes

Our main outcome is the mediation effect of exercise-induced myokine level changes on exercise-induced cognitive function changes in older adults. To be eligible for inclusion, studies must report both the myokine and the cognition outcomes.

A total of 1,126 secretory proteins, identified as putative myokines, by definition secreted by skeletal muscle and exerting a biological function in a paracrine or endocrine fashion (Whitham and Febbraio, 2016), have been compiled from existing literature. A comprehensive list, along with corresponding references, can be found in Supplementary Appendix B. The primary sources for this compilation of myokines include proteomic analysis, secretome analysis, and mRNA sequencing studies on human skeletal muscle (Bortoluzzi et al., 2006; Hittel et al., 2009; Norheim et al., 2011; Le Bihan et al., 2012; Raschke et al., 2013; Scheler et al., 2013; Catoire et al., 2014; Hartwig et al., 2014; Pourteymour et al., 2017), some of which specifically studied myokines that are elevated in response to exercise or muscle contraction (Norheim et al., 2011; Raschke et al., 2013; Scheler et al., 2013; Catoire et al., 2014; Pourteymour et al., 2017) and review papers (Engler, 2007; Catoire and Kersten, 2015; Görgens et al., 2015; Kwon et al., 2020; Rai and Demontis, 2022). Notably, lactate and beta-aminoisobutyric acid (BAIBA), categorized most often as myometabolites, but recently also as myokines due to their endocrine effects will also be included (Catoire and Kersten, 2015; Brooks et al., 2023). Additionally, the enzyme kynurenine aminotransferase and kynurenine-derived metabolites have also been referred to as myokines, despite kynurenine itself being produced by the liver, while kynurenine aminotransferase is found on muscle cells (Rai and Demontis, 2022). By employing this inclusive approach, our objective is to generate a comprehensive list of myokines that may potentially impact cognitive function in older adults. However, it is important to acknowledge that for the majority of these putative myokines limited research has been conducted on the exercise-cognition context specifically. Moreover, there exists a lack of comprehensive understanding regarding the biological effects of most discovered putative myokines in general (Lee and Jun, 2019), and most of the myokines likely exert paracrine, not endocrine effects (Weigert et al., 2014). Consequently, we anticipate that our systematic search will likely yield only a concise compilation of myokines relevant to the exercise-cognition field, considering the current state of knowledge. It should be noted that the included putative myokines are allowed to be also released partly from other organs, as long as part of the exercise-induced changes (increase or decrease) in levels in the bloodstream is caused by pathways activated in skeletal muscle tissue. We will exclude myokines that are not released from muscle tissue, even if they have a known effect on the brain, such as growth hormone (GH), Orexin-A, or Ghrelin [see review paper of Vints et al. (2022b) where a schematic overview of exerkines with known effects on neuroplasticity in aerobic and resistance exercise is described]. Myokine levels measured in total blood, blood serum, blood plasma, or cerebrospinal fluid will be included.

Concerning cognition, we will include intrinsic capacity domains that are particularly vulnerable in older age (fluid cognitive functions, as opposed to crystallized intelligence) based on the Cattell-Horn-Carroll-Miyake taxonomy of cognitive domains (Webb et al., 2018).

The cognitive domains include fluid reasoning (e.g., Raven’s progressive matrices), long-term memory (e.g., Rey Auditory Verbal Learning test), short-term memory (e.g., Complex span tasks and Digit span backwards), executive functions (e.g., N-back, Dual-tasking, and Stroop test), processing speed (e.g., Digit symbol substitution task and Choice reaction time), visual processing (e.g., Visual search), global cognitive functioning [e.g., mini-mental state examination (MMSE), Montreal Cognitive Assessment (MoCA), Cognitive Failures Questionnaire (CFQ)]. Cognitive measures will include response time (of all or only the correct answers), throughput (of all or only the correct answers), performance index (100 × [accuracy/response time]), accuracy (number or % correct answers), or specific test scores.

#### 2.1.6. Timing of outcome assessments after end of intervention

Concerning the timing of post-exercise assessment, it is critical to distinguish between acute and chronic exercise effects. Any measurement immediately after a single bout of exercise (irrespective of the time period since the last exercise bout), is generally considered an acute exercise effect. However, in order to measure chronic exercise effects after multiple bouts of exercise, it is advised to measure the outcome more than 24 h, but preferably more than 48 h after the last exercise session (Vints et al., 2022b). If outcome assessments were conducted less than or equal to 24 h after the last exercise bout of an intervention consisting of multiple exercise bouts, we will consider them acute exercise effects in trained individuals. If outcome assessments were conducted more than 24 h after exercise, we will consider them chronic exercise effects. The timing of the outcome assessment will not be a criterion for inclusion of a study, but the information will be extracted, and the decision whether it is considered acute or chronic effect, will be taken accordingly.

#### 2.1.7. Setting

Included studies will not be restricted to a specific type of setting. Expected settings include community settings (e.g., day care centers, universities and workplace), clinical settings (e.g., hospitals and psychiatric institutions), and home settings (e.g., people’s own home, nursing homes, and care homes).

#### 2.1.8. Language

We will only include articles written in the English language.

### 2.2. Information sources

The authors [Wouter A. J. Vints and Ioanna Zorba (Zormpa) – information specialist] will search in PubMed, EMBASE (through Elsevier), PsycINFO (through EBSCO), all databases of Web of Science (excluding MEDLINE), SportDiscus (through EBSCO), LILACS (accessed through Portal Regional da BVS), IBECS (accessed through Portal Regional da BVS), CINAHL (through EBSCO), SCOPUS (Elsevier), International Clinical Trials Registry Platform (ICTRP) accessed through CENTRAL, and ClinicalTrials.gov (CT.gov) accessed through CENTRAL. None of the databases will be restricted by date. The searches will be re-run prior to the final analysis.

### 2.3. Search details

Literature search strategies are developed using free text words and index terms related to: middle or older age AND physical exercise AND cognition AND myokines. Supplementary Appendix A includes a transcript of the free terms used in the search strategy and the index terms per database where applicable. The search strategy was translated for each of the used databases in a way that they remained as equal as possible, using the same free text terms, and searching for the most similar index terms. The search strategy was developed by Wouter A. J. Vints, who has expertise in the topic of the review with the help of Ioanna Zorba (Zormpa) who works as an information specialist, and was approved by all collaborating authors. Details of the used limitations and filters are provided in Table 1.

**TABLE 1:** Details of limitations and filters per database.

Database	Limitations/filters
PubMed	Free terms were searched in the fields of title, abstract, and author keywords Limitations/filters: randomized controlled trial, humans, English, exclude preprints, middle aged OR aged: 45+ years
Embase	Free terms were searched in the fields of title, abstract, and author keywords Limitations/filters: randomized controlled trial, human, aged OR middle aged OR very elderly
SportDiscus	Free terms were searched in the fields of title, abstract, and author keywords Limitations/filters: English
PsycInfo	Free terms were searched in the fields of title, abstract, and author keywords Limitations/filters: English, population group: human, exclude dissertations, age groups: middle age (40–64 years) OR aged (65 years and older) OR very old (85 years and older)
LILACS	Free terms were searched in the fields of title, abstract, and subject Limitations/filters: English
IBECS	Free terms were searched in the fields of title, abstract, and subject Limitations/filters: English
Scopus	Free terms were searched in the fields of title, abstract, and author keywords Limitations/filters: randomized controlled trial, English
CINAHL	Free terms were searched in the fields of title, abstract, and topic Limitations/filters: English, human, exclude MEDLINE records, middle aged OR aged OR aged 80 and over
Web of Science	Free terms were searched in the fields of title, abstract, and indexing Limitations/filters: English, article, all databases excluding MEDLINE records
ClinicalTrials.gov	Free terms were searched in the fields of title, abstract, and author keywords Limitations/filters: English, trial
International Clinical Trials Registry Platform	Free terms were searched in the fields of title, abstract, and author keywords Limitations/filters: English, trial

### 2.4. Data management (search management and data extraction)

#### 2.4.1. Study selection

Pairs of two authors will be involved in the study selection process. Due to the expected large amount of studies, the workload will be divided between all authors. Two authors will independently, without knowing each other’s decisions, screen the eligibility of the studies with a specific publication date, while studies with other publication dates will be screened by different pairs of authors. The first screening step will be based on the title and abstract of the article and in a second step based on the full text article. If the two authors disagree at any step, a third author will evaluate the eligibility criteria and debate among all three authors will be organized to reach an agreement. The software system “Rayyan” will be used for the study selection process (Ouzzani et al., 2016).

#### 2.4.2. Data extraction

Following the selection process, pairs of two authors will independently extract the following information:

– The name of the first author
– Year of publication
– Study design
– Participant characteristics [number in each group, age (range and mean), sex (%), BMI (range and mean), level of education, physical fitness, main clinical diagnosis, and comorbidities]
– Exercise characteristics [type of exercise, intensity, duration in weeks or months, frequency, length of one exercise session, follow-up time, home-based vs. gym-based, autonomous vs. with a trainer (specify the type of trainer, e.g., researcher and professional coach)]
– Control group characteristics (type of control condition)
– Cognition (cognitive domain assessed, cognitive test used, how was the outcome measured, e.g., as response speed – accuracy – performance index – throughput – specific test score, whether a test was performed by a professional, e.g., psychiatrist – nurse – trained personnel – untrained personnel)
– Acute effect in trained/untrained individuals, or chronic effect (how long after the last exercise bout was the cognitive outcome measured and the blood collection performed)
– Sample size in treatment and control group
– Standardized mean differences (SMDs) between control and treatment group on myokines (or the necessary statistics to calculate SMDs)
– SMDs between control and treatment group on cognitive functioning (or the necessary statistics to calculate SMDs)
– Bivariate correlations between myokines and cognitive functioning (preferably in the total sample, or else per condition)
– Bivariate correlations between different types of myokines (preferably in the total sample, or else per condition)
– Bivariate correlations between different types of cognitive functioning (preferably in the total sample, or else per condition).

The exercise characteristics will additionally be used to place the studies in specific categories for subgroup analyses. We will extract information about the type of exercise: cardiorespiratory endurance exercise (such as walking, running, or cycling aimed to improve the aerobic energy systems), high-intensity interval training (including sprint training or other interval-based cardiorespiratory exercise training aimed to improve both aerobic and anaerobic energy systems), resistance exercise (such as weight lifting, strength training, power training, body weighted exercises, and elastic bands exercises intended to increase muscular strength/volume/power), mind-body exercises (such as Tai Chi, yoga, motor skill training, or dance aimed to improve mind-body coordination and awareness by a series of controlled movements that focus on the interactions between the brain, body, mind, and behavior), balance training (aimed to improve balance or proprioception), multicomponent exercise (i.e., a combination of at least two of the aforementioned types of exercise), or other specific exercises (e.g., basketball training or competition) that do not fit in any of these categories. We will also extract information about the, intensity of exercise (very light, moderate, vigorous, or near-maximal to maximal intensity based on the American College of Sports Medicine Position Stand, published July 2011 by Garber et al. (2011) in Medicine and Science in Sports and Exercise), the intervention duration in weeks (months) [single bout, <13 weeks (3 months), 13–26 weeks, and >26 weeks (6 months)], the frequency [single bout, <3, 3–5, and >5 times/week], and the exercise session length (<30, 30–60, and >60 min).

Participant characteristics will also be extracted to be used in subgroup analysis, including age groups (50–69 years old vs. 70 years and over), sex, healthy vs. disease, normal cognition vs. cognitive impairment at baseline, fitness level at baseline, and educational level.

In case of disagreement in the extracted information, a third author will be requested to evaluate the data and debate among all three reviewers will be organized to reach an agreement. In case of missing information, the study investigators will be contacted. When no contact details are available or no reply is received within 1 month, the missing information will be marked “not available.”

### 2.5. Risk of bias assessment

Risk of bias will be assessed using the Cochrane Risk of Bias tool (ROB 2) for randomized controlled trials (Higgins et al., 2011). The possible risk of bias on each of the domains included in these risk of bias tools will be judged as “high risk,” “low risk” or “unclear.” The individual results will be compared between two review authors and disagreements will be solved by consulting a third author for arbitration.

### 2.6. Data synthesis and statistical methods

#### 2.6.1. Meta-analysis with MASEM

If data is appropriate for synthesis, we will conduct a meta-analysis using random effects meta-analyses. We will assess the effect of exercise on each of the myokines and each of the cognitive functioning outcomes that are included in at least three or more included randomized controlled trials.

**FIGURE 1:**
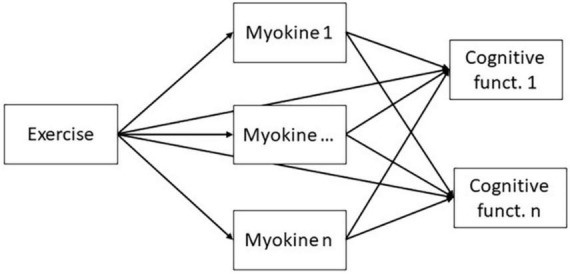
Meta-analytic structural equation modeling (MASEM) model.

A path model (see Figure 1) will be evaluated using one-stage meta-analytic structural equation modeling (MASEM). One-stage MASEM is essentially a random-effects multivariate meta-analysis on correlation coefficients, in which the average correlations are restricted to follow the structure of the hypothesized path model. In its simplest form, the random-effects multivariate meta-analytic model decomposes the vector *r*_*k*_ of correlation coefficients for a study *k* in three parts:


(1) $$r_{k}=\rho_{R}+u_{k}+\varepsilon_{k},$$

where *ρ_*R*_* indicates the vector of means of the correlation coefficients over all studies, *u*_*k*_ is a vector of deviations of study *k*’s population correlation coefficients from *ρ_*R*_*, and *ε_*k*_* is a vector with the sampling deviations of study *k*. The covariances of *u*_*k*_ denote the between-studies covariance matrix. The covariances of *ε_*k*_* represents the within-studies covariance matrix for study *k*, often denoted *V*_*k*_, which is estimated for each study, and then treated as known in the final analysis. Estimates of the path coefficients are obtained by restricting *ρ_*R*_* in Equation 1:


(2) $$\rho_{R}=\text{vechs}\,\left(\text{F}\,{\left(\text{I}-\text{A}\right)}^{-1}\,\text{S}\,{\left(\text{I}-\text{A}\right)}^{-1\,T}\,{\text{F}}^{T}\right),$$

where using the RAM-formulation (McArdle and McDonald, 1984), **I** is an identity matrix, **F** is a selection matrix with 1’s for observed variables and 0’s for latent variables, **A** is a square matrix with asymmetric paths such as regression coefficients, **S** is a symmetrical matrix with variances and covariances, and vechs() vectorizes the lower diagonal of its argument. For more explanation and details of this method see Jak and Cheung (2020) and Jak et al. (2021).

This process will be led by co-author Suzanne Jak. The analyses will be conducted using the metaSEM package in R (Cheung, 2015).

Since one-stage MASEM uses correlation coefficients as input, the SMDs on myokines and cognitive functioning will be transformed to point-biserial correlations with a target base-rate of 0.50 (see McGrath and Meyer, 2006). The MASEM will lead to estimates of each of the direct effects in the path model, as well as estimates of the indirect effects of exercise on cognitive functioning through the different myokines. Statistical significance of direct effects will be evaluated using Wald-tests with a significance level of 5%, and statistical significance of mediating (indirect) effects with 95% likelihood-based confidence intervals (Neale and Miller, 1997; Cheung, 2022). A comparison of the strength of effects for different types of myokines and cognitive functions will be performed by constraining the relevant parameters to be equal and evaluating the difference in model’s log likelihoods.

Studies on acute exercise effects will be analyzed separately from chronic exercise studies. As a sensitivity analysis, we will also execute random-effects meta-analysis on the SMDs of myokines and cognitive outcomes separately using “standard” (not SEM) meta-analysis. If there are dependent effect sizes within studies (e.g., multiple operationalization of the same construct) they will be taken into account using robust variance estimation (Hedges et al., 2010).

#### 2.6.2. Heterogeneity

In order to try to explain possible heterogeneity across studies, we will evaluate moderating effects on all path coefficients in the model for all moderators that have enough coded values in studies. The moderating effects will be evaluated separately for each moderator. Moderators under consideration include exercise characteristics such as type of exercise, intensity of exercise, intervention duration in weeks, frequency of exercise sessions per week, exercise session length, participant characteristics including age groups, sex, health status, normal cognition or cognitive impairment at baseline, fitness level at baseline, and educational level.

#### 2.6.3. Meta-biases

The potential for reporting bias and publication bias will be explored by visual inspection of funnel plots of the SMDs if ≥10 studies are available. We will also perform Egger’s regression tests on the SMD’s.

#### 2.6.4. Quality of evidence

The quality of evidence for all outcomes will be judged using the Grading of Recommendations Assessment, Development, and Evaluation (GRADE) working group methodology (Akl et al., 2013). The quality of evidence will be assessed across the domains of risk of bias, consistency, directness, precision, and publication bias. Additional domains may be considered where appropriate. Quality will be adjudicated as high (further research is very unlikely to change our confidence in the estimate of effect), moderate (further research is likely to have an important impact on our confidence in the estimate of effect and may change the estimate), low (further research is very likely to have an important impact on our confidence in the estimate of effect and is likely to change the estimate), or very low (very uncertain about the estimate of effect). The effect of study quality on the effect sizes will be evaluated using meta-regression.

### 2.7. Administration, dissemination, and updating of the living review

We plan to update the systematic review and meta-analysis for at least 5 years, with the option to extend this period if agreed by the authors. However, we may choose to convert the paper from a living systematic review to a normal systematic review and meta-analysis if the authors collectively determine that the criteria for conducting a living systematic review are no longer applicable. This may occur if there is no longer a significant level of uncertainty in the existing evidence, or if the research field is no longer rapidly evolving with emerging evidence likely to impact the conclusions of the living systematic review.

The project will be managed by one author (Wouter A. J. Vints). We plan to rerun the searches every 6 months and search for new myokines that are not yet included in the review on a yearly basis. The searches will be managed by one author (Wouter A. J. Vints) with the support of a librarian [Ioanna Zorba (Zormpa)] upon request. The division of other review tasks will remain as decided for the first review process. Whenever a contributing author wishes to step out of the review update process, he/she will try his/her best to assign a replacement or the replacement will be decided by the other contributing authors.

The decision to update the review paper will be based on a published scheme (Elliott et al., 2017). Updates of the living systematic review and meta-analysis that do not require re-publication will be presented at the website, https://www.egrepa.org/. If no new studies are found, only the date of the last search will be updated. If new studies are found, but the new information is likely to have negligible effects on the effect estimates or the certainty of the evidence, the date of the last search will be updated together with details of the new evidence and a justification for delaying the incorporation of this evidence in the paper. If new studies are found with likely effects on the effect estimates or the certainty of evidence, the date of the last search will be updated, the details of the new evidence will be added and data will be extracted, quality will be assessed and synthesized and the studies will immediately be incorporated in the paper. Once this process is completed, the updated living systematic review and meta-analysis will be submitted for re-publication in an open access international peer-reviewed journal as soon as possible, preferably a journal that has the resources to support a living systematic review. A summary is presented in Table 2.

**TABLE 2:** Intended process and publication strategy of the living review.

Number of authors maintaining the LSR	15
Search support	Information specialist within the author team [Ioanna Zorba (Zormpa)]
Search frequency	Every 6 months
Communicating review status to reader	On the PhysAgeNet website, the EGREPA website and via an article amendment if the latter is allowed by the journal and its editorial. https://physagenet.eu/ https://www.egrepa.org/
Process for integration of new evidence	Full re-publication of the review, with new citation and DOI whenever new information is retrieved that will likely affect the effect estimates or the certainty of evidence.
Trigger for integration of new evidence	Every 12–24 months depending on the impact of the new evidence, according to the decision tree presented by Elliott et al. (2017).

## 3. Discussion

### 3.1. Perspectives

This proposed systematic review paper with meta-analysis is expected to make a significant contribution to the existing literature by generating new and valuable knowledge.

Firstly, this will be the first meta-analysis to comprehensively investigate the triad relationship between exercise, myokines and cognition in older adults. The analysis will not be restricted to specific types of exercise, health status, sets of myokines or cognitive function domains. We will use advanced statistical methods that have not been applied in this topic before to assess whether specific myokines mediate the relationship between exercise and cognition.

Secondly, the living mode of this review paper allows for continuous updates, to ensure that the information remains up-to-date and relevant. As the research field progresses, the review will include the latest findings and become a valuable resource for researchers, clinicians, and policymakers.

Lastly, this review paper could have extensive practical implications by providing insights into which myokines are critical for maintaining cognitive health in older adults.

As the review progresses, it may provide more specific information on the value of certain myokines for enhancing particular cognitive domains or treating specific diseases. In addition, we will continuously update the review with information on the optimal exercise characteristics for inducing these myokines such as the mode of exercise, intensity, and other factors. Ultimately, this could help us to individualize exercise programs to meet the specific needs of older adults. The insights gained from this review may extend beyond older adults, providing broader benefits to society. In the future, this knowledge may lead to the design of healthy aging interventions, and even lead to the development of myokine-containing pharmacological pills. Such interventions could be used as add-ons or to mimic the effect of exercise for those unable to participate in optimal exercise interventions (see Gubert and Hannan, 2021).

### 3.2. Strengths and limitations

One important strength of our study design is its living approach, which enables us to keep this review paper up to date over time. Notably, research has shown that 25% of systematic reviews lose their accuracy and utility within 2 years (Shojania et al., 2007). An important limitation of most systematic reviews, especially in a rapidly growing research field like the one we are studying, is that they often become outdated quickly. In contrast, our review paper targets a wide range of studies, without restricting our scope to a specific set of myokines, including 1,126 myokines at start, cognitive outcomes, exercise interventions or population criteria, except for older age. This approach will provide a comprehensive overview of the research field. Additionally, we will use advanced statistical techniques such as MASEM, to examine the mediating effects of myokines on cognitive changes. This information may drastically change the understanding of the role of myokines in cognitive function changes in older adults with varying health status following exercise.

The limitations of our study design include the expected retrieval of a highly heterogeneous list of studies, which is a common issue in meta-analyses conducted in this research field. The reasons behind this variability include differences in participant characteristics, exercise protocols, blood sampling methods, and cognitive tests used (Vints et al., 2022b). Furthermore, we made a conscious decision to impose fewer restrictions on the inclusion of studies in order to provide a comprehensive overview of the field. However, this decision may increase the heterogeneity of the included studies, which is a potential limitation of our design. In addition, it should be noted that some studies use low-intensity exercise or balancing exercise as a control condition in their research, but we have decided not to include studies with an exercise control group in our analysis. This decision also constitutes a limitation of our study design. Finally, our search will be limited to the English literature, which could be considered a form of bias. However, this is common practice, as only one third of systematic reviews report to conduct searches without language restriction and only 2% eventually include non-English literature (Jackson and Kuriyama, 2019; Pieper and Puljak, 2021).

## 4. Conclusion

This protocol outlines the methods for a living systematic review with meta-analysis, which aims to investigate the role of more than 1,125 putative myokines as potential mediators in the relationship between exercise and cognitive function in older adults. The existence of a direct cross-talk between muscle and brain via myokine signaling has been demonstrated, indicating the potential for muscle-derived signaling factors to be used as a non-pharmacological intervention to maintain cognitive ability at older age. However, the rapid growth of this research field necessitates continued synthesis to identify the most promising targets and exercise protocols. A living systematic review with meta-analysis can facilitate ongoing research into the specific bioactivity of myokines and their association with cognitive function, leading to a better understanding of the relationship between exercise and cognitive health in older adults.

## Author contributions

WV, YN, EG, and AL wrote the first draft of this protocol. WV and IZ developed the search strategies. SJ developed and described the statistical methods. All authors reviewed and approved the final version.

## Footnotes

1
https://www.cost.eu/actions/CA20104/
