# Supplementary material for: Myokines as mediators of exercise-induced cognitive changes in older adults: protocol for a comprehensive living systematic review and meta-analysis
Source: Front Aging Neurosci. 2023 Jul 13;15:1213057. doi: 10.3389/fnagi.2023.1213057 (PMC10374322; doi:10.3389/fnagi.2023.1213057)
Supplement: Supplementary file 2 [file Table_1.docx]

Supplementary Material

**Myokines as Mediators of Exercise-Induced Cognitive Changes in Older Adults: Protocol for a Comprehensive Living Systematic Review and Meta-Analysis**

**Wouter A.J. Vints MD^*^, Evrim Gökçe PhD, Antoine Langeard PhD, Iuliia Pavlova PhD, Özge Selin Çevik PhD, Mohammad Mosaferi Ziaaldini PhD, Jasemin Todri PhD, Orges Lena PhD, Giorgos K. Sakkas PhD, Suzanne Jak PhD, Ioanna Zorba (Zormpa) PhD, Christina Karatzaferi PhD, Oron Levin PhD, Nerijus Masiulis PhD, Yael Netz PhD**

*** Correspondence:**

Wouter Arthur Johan Vints, MD

w.vints@maastrichtuniversity.nl

**1. Appendix A Search strategy**

Free terms for all databases:

("Middle Aged" OR "older adult*" OR "older age*" OR "older individual*" OR "older men" OR "older patient*" OR "older people" OR "older people*" OR "older person*" OR "older population*" OR "older women" OR Aged OR ageing OR Aging OR elder* OR geriatric* OR senescence OR senior*)

AND

("mind body training" OR "mind-body training" OR "motor activity" OR "muscle activity" OR "muscle training" OR "occlusion training" OR "physical activit*" OR "physical condition*" OR "physical exertion" OR "physical rehabilitation" OR "Physical Therapy" OR "physical training" OR "power training" OR "qi gong" OR "resistance training" OR "stability training" OR "strength training" OR "tai chi" OR "tai ji" OR "weight lift*" OR "weight training" OR "Wingate test" OR baseball OR basketball OR bicycl* OR biking OR box OR boxe* OR boxi* OR cricket OR cycl* OR danc* OR Exercis* OR exergam* OR feldenkrais OR football OR golf* OR gym OR gymn* OR hiking OR hockey OR jog OR jogg* OR judo OR kaatsu OR Kinect OR pilates OR plyometric OR rugby OR run OR runn* OR skating OR skiing OR soccer OR sport* OR sprint* OR swim* OR Tennis OR volleyball OR walk* OR Weightlifting OR Wii OR wrestl* OR yoga OR "active video gam*" OR "aerobic training" OR "anaerobic training" OR "balance training" OR "blood flow restriction training" OR "body training" OR "cardiac rehabilitation" OR "cardiorespiratory training" OR "cardiovascular training" OR "circuit training" OR "concentric training" OR "eccentric training" OR "endurance training" OR "Exercise Test" OR "Exercise Therapy" OR "high intensity interval training" OR "high-intensity interval training" OR "hypertrophy training" OR "isokinetic training" OR "locomotor activity" OR "Martial Arts")

AND

("decision making" OR "dual task*" OR "Mental Processes" OR "Mini Mental State Examination" OR "neuropsychological assessment" OR "problem solving" OR "psychomotor speed" OR "reaction time*" OR "response time*" OR "Simon task" OR "Symbol Digit Modalit*" OR "task switching" OR "Tower of Hanoi" OR "Tower of London" OR "Trail Making" OR "Verbal Fluency" OR "visual test" OR "visuospatial test" OR "Wisconsin Card Sorting" OR "Word Association" OR accuracy OR Attention OR cogniti* OR executive OR Flanker OR inhibition OR language OR learning OR memory OR memor* OR mental OR MMSE OR MoCA OR neurocognition OR neurocogni* OR neuropsychologic* OR oddball OR planning OR processing OR reasoning OR recall OR recogniti* OR retention OR Sternberg OR Stroop OR WAIS OR Wechsler)

AND

("myokine*" OR "miokine*" OR "heat shock protein" OR "selenoprotein" OR "17-beta-hydroxysteroid dehydrogenase type VII-like" OR "17-β-hydroxysteroid dehydrogenase type VII-like" OR "3',5'-bisphosphate nucleotidase*" OR "2',5'-bisphosphate nucleotidase*" OR "40S ribosomal protein S10" OR "40S ribosomal protein S19" OR "40S ribosomal protein S28" OR "40S ribosomal protein S4" OR "40S ribosomal protein SA" OR "calcium-binding protein" OR "6-phosphogluconolactonase" OR "collagenase" OR "glucose-regulated protein" OR "abhydrolase domain-containing protein*" OR "acetylgalactosaminyltransferase" OR "galactosyltransferase" OR "actin" OR "acylamino-acid-releasing enzyme" OR "ADAM 9" OR "ADAM-9" OR "ADAM*" OR "ADAM metallopeptidase" OR "ADAM-like" OR "ADAMTS-like*" OR "adenosylhomocysteinase" OR "adenylate cyclase activating polypeptide 1 receptor" OR "Adenylate kinase isoenzyme*" OR "adipocyte enhancer binding protein*" OR "adiponectin*" OR "Adlican" OR "adrenomedullin" OR "advanced glycosylation end product-specific receptor" OR "AE binding protein 1" OR "pancreas tumor-related protein" OR "aggrecan" OR "agrin" OR "aldehyde dehydrogenase mitochondrial" OR "aldo-keto reductase family 1 member C3" OR "Alkaline phosphatase" OR "Alpha 1 type I collagen" OR "Alpha 1 type 1 collagen" OR "α1 type I collagen" OR "α1 type 1 collagen" OR "Alpha 1 type III collagen" OR "Alpha 1 type IV collagen " OR "Alpha 1 type VIII collagen " OR "Alpha 1 type XVIII collagen isoform 1 " OR "Alpha 2 macroglobulin " OR "Alpha 2 type I collagen" OR "Alpha 2 type IV collagen " OR "Alpha 3 type VI collagen isoform 1 " OR "alpha-2-macroglobulin" OR "alpha-crystallin" OR "alpha-enolase" OR "Alpha-fetoprotein " OR "alpha-mannosidase 2" OR "alpha-soluble NSF attachment protein" OR "α1 type III collagen" OR "α1 type IV collagen " OR "α1 type VIII collagen " OR "α1 type XVIII collagen isoform 1 " OR "α2 macroglobulin " OR "α2 type I collagen" OR "α2 type IV collagen " OR "α3 type VI collagen isoform 1 " OR "α2-macroglobulin" OR "α-crystallin" OR "α-enolase" OR "α-fetoprotein " OR "α-mannosidase*" OR "α-soluble NSF attachment protein" OR "aminopeptidase N" OR "Amplified in osteosarcoma" OR "Amylase" OR "amyloid beta A4 protein" OR "amyloid-β A4 protein" OR "Aβ A4 protein" OR "Angiogenin" OR "Angiopoietin*" OR "angiotensin I converting enzyme" OR "angiotensin 1 converting enzyme" OR "Angiotensinogen" OR "ankyrin repeat and SOCS box containing" OR "annexin*" OR "Antithrombin-III" OR "apelin" OR "Apolipoprotein" OR "Arylsulfatase A" OR "arylsulfatase family member I" OR "Aspartate aminotransferase" OR "asporin" OR "astrocytic phosphoprotein" OR "ATP synthase subunit beta" OR "ATP-citrate synthase" OR "basal cell adhesion molecule" OR "basement membrane-specific heparan sulfate proteoglycan core protein" OR "Basement Membrane Protein*" OR "BBP-like protein*" OR "beta actin like protein 2" OR "β-actin like protein 2" OR "acetylglucosaminyltransferase lunatic fringe" OR "galactosyltransferase*" OR "beta-2-microglobulin" OR "β2-microglobulin" OR "beta-aminoisobutyric acid" OR "beta-hexosaminidase" OR "beta-mannosidase" OR "Beta-microseminoprotein" OR "β-aminoisobutyric acid" OR "β-hexosaminidase" OR "β-mannosidase" OR " mannosidase-β" OR "mannosidase beta" OR "β-microseminoprotein" OR "biglycan" OR "Biotinidase " OR "Bone morphogenetic protein*" OR "Bone morphogenetic factor*" OR "brain-derived neurotrophic factor" OR "BTB domain-containing protein" OR "POZ domain-containing protein" OR "butyrophilin" OR "C type lectin domain family 11 member A" OR "Complement C1q " OR "cadherin*" OR "calcitonin receptor" OR "calcyclin" OR "calmodulin" OR "calreticulin" OR "Calsyntenin*" OR "Calumenin" OR "carbonyl reductase*" OR "carboxylesterase*" OR "Carboxypeptidase* " OR "carcinoembryonic antigen-related cell adhesion molecule*" OR "Carnosinase*" OR "Cartilage intermediate layer protein" OR "cartilage oligomeric matrix protein" OR "Cat eye syndrome chromosome region" OR "cathepsin*" OR "C-C motif*" OR "chemokine*" OR "CD109 antigen " OR "CD163 molecule-like*" OR "CD200 molecule" OR "CD300 molecule" OR "CD44 antigen" OR "CD99 antigen-like 2" OR "C-X3-C motif" OR "C-X-C motif" OR "Chitinase*" OR "chloride intracellular channel protein*" OR "chondroadherin*" OR "acetylgalactosaminyltransferase*" OR "Chondroitin sulfate" OR "versican" OR "Chordin-like 1" OR "choriogonadotropin" OR "chromogranin B" OR "ciliary neurotrophic factor receptor" OR "clusterin*" OR "coactosin-like protein" OR "coagulation factor" OR "thrombin" OR "coatomer subunit delta" OR "cofilin*" OR "coiled-coil domain*" OR "collagen alpha*" OR "collagen triple helix repeat containing*" OR "collagen α*" OR "colony stimulating factor*" OR "C1q tumor necrosis factor-related protein" OR "Complement C1r" OR "complement C1s" OR "complement C4-A" OR "complement C4A" OR "Complement C1 inhibitor" OR "Complement C1r-like" OR "Complement C2" OR "Complement C3 " OR "Complement C4 binding protein" OR "Complement C4-B" OR "Complement C4B" OR "Complement C5" OR "Complement C7 " OR "complement C8" OR "Complement factor B" OR "Complement factor D" OR "Complement factor H" OR "complement factor properdin" OR "connective tissue growth factor" OR "contactin associated protein-like" OR "corticotropin releasing hormone receptor" OR "C-reactive protein " OR "C-type lectin domain family 11 member A" OR "C-type lectin domain family 2 member D" OR "C-type mannose receptor 2" OR "EGF-like repeat containing 3" OR "CUB containing protein 3" OR "Cubilin" OR "Cystatin S" OR "Cystatin-C" OR "Cysteine knot superfamily 1" OR "BMP antagonist 1" OR "cysteine rich angiogenic inducer" OR "Cysteine-rich secretory protein" OR "cysteine-rich secretory protein" OR "cytochrome P450 family 2" OR "cytochrome p450 oxidoreductase" OR "Cytokine*" OR "decorin" OR "Defensin" OR "dehydrogenase/reductase (SDR family)" OR "Deleted in malignant brain tumors" OR "aminolevulinic acid dehydratase" OR "delta-like 1" OR "Deoxyribonuclease II" OR "Deoxyribonuclease 2" OR "dermcidin" OR "desmin" OR "destrin" OR "DFNA5" OR "deafness associated tumor suppressor" OR "Dickkopf homolog" OR "Dickkopf-related protein" OR "dihydrolipoyl dehydrogenase" OR "dipeptidyl peptidase" OR "disintegrin and metalloproteinase domain-containing protein" OR "DKFZP564O0823 protein" OR "DKFZP586H2123 protein" OR "DKFZP586M0622 protein " OR "DNA damage-binding protein" OR "DnaJ heat shock protein family" OR "dual specificity phosphatase" OR "dual specificity tyrosine phosphorylation regulated kinase" OR "dystroglycan" OR "Ectonucleotide pyrophosphatase" OR "Ectonucleotide phosphodiesterase" OR "EGF-containing fibulin-like extracellular matrix protein" OR "EGF-like repeat and discoidin I-like domain-containing protein" OR "EGF-like repeat and discoidin 1-like domain-containing protein" OR "EGF-like repeats and discoidin I-like domains-containing protein" OR "EGF-like repeats and discoidin 1-like domains-containing protein" OR "ElaC homolog 2" OR "elastin" OR "elastin microfibril interfacer" OR "elongation factor" OR "EMILIN" OR "enamelin" OR "Endoplasmic reticulum aminopeptidase" OR "endoplasmic reticulum resident protein" OR "endoplasmin" OR "endosialin" OR "Endothelial lipase" OR "endothelin" OR "enolyl-CoA delta isomerase 1" OR "Eotaxin" OR "EPH receptor A2" OR "EPH receptor A2" OR "EPH receptor A3" OR "EPH receptor A3" OR "EPH receptor B1" OR "ephrin type-A receptor 4" OR "Epididymis-specific alpha-mannosidase" OR "erb-b2 receptor tyrosine kinase 3" OR "Erythrocyte membrane protein band" OR "ES1 protein homolog" OR "eukaryotic translation initiation factor" OR "Exostosin-like 2" OR "Extracellular matrix" OR "Extracellular superoxide dismutase" OR "ezrin" OR "F2R like thrombin/trypsin receptor 3" OR "F-actin-capping protein subunit beta" OR "family with sequence similarity" OR "farnesyl pyrophosphate synthetase" OR "FAT atypical cadherin" OR "IgG Fc binding protein" OR "Fc fragment of IgG" OR "ferritin heavy chain" OR "Fibrillin*" OR "Fibrinogen" OR "Fibroblast growth factor*" OR "Fibrocystin*" OR "Fibromodulin*" OR "fibronectin*" OR "fibulin*" OR "ficolin*" OR "FK506-binding protein*" OR "FLJ21919 protein" OR "fms-related tyrosine kinase*" OR "Follicular lymphoma variant translocation*" OR "follistatin*" OR "four and a half LIM domains protein*" OR "four jointed box*" OR "fractalkine" OR "frizzled-related protein" OR "fucosyltransferase*" OR "G protein-coupled receptor 162" OR "galactosidase alpha" OR "Galectin*" OR "gamma-aminobutyric acid type A receptor" OR "gamma-enolase" OR "Gamma-glutamyl hydrolase" OR "Gamma-glutamylcyclotransferase" OR "Gamma-interferon-inducible*" OR "gelsolin" OR "GK001 protein" OR "nexin" OR "glial cell derived neurotrophic factor" OR "Glioblastoma amplified sequence" OR "Glucose-6-phosphate isomerase" OR "glucosidase 2" OR "glutamate metabotropic receptor" OR "glutamate receptor 2C" OR "glutamine-fructose-6-phosphate transaminase*" OR "glutaminyl-peptide cyclotransferase" OR "Glutathion peroxidase*" OR "Glutathione reductase" OR "glutathione S-transferase P" OR "glycoprotein hormones, alpha polypeptide" OR "glycyl-tRNA synthetase" OR "glyoxalase domain-containing protein*" OR "glypican*" OR "Gonadotropin-releasing hormone*" OR "Granulin*" OR "granulocyte chemotactic protein*" OR "granulocyte colony-stimulating factor" OR "granulysin" OR "gremlin*" OR "Growth arrest-specific*" OR "Growth/differentiation factor*" OR "Growth differentiation factor*" OR "growth-regulated alpha protein" OR "GTP-binding nuclear protein Ran" OR "guanylate binding protein*" OR "H factor 1" OR "HB-EGF" OR "heme-binding protein" OR "hemoglobin subunit alpha 2" OR "heparan sulfate proteoglycan*" OR "Heparin-binding growth factor*" OR "Hepatocyte growth factor" OR "Hepatoma-derived growth factor" OR "hephaestin" OR "hexose-6-phosphate dehydrogenase" OR "glucose 1-dehydrogenase" OR "Histatin*" OR "Histone H2A" OR "histone H3" OR "histone-lysine N-methyltransferase" OR "HNOEL-iso protein" OR "Homolog of zebrafish ES1 isoform" OR "Hyaluronan and proteoglycan link protein*" OR "Hyaluronan binding protein*" OR "hyaluronan synthase*" OR "hydroxyacylglutathione hydrolase" OR "hydroxysteroid 17-beta dehydrogenase*" OR "I factor" OR "interferon*" OR "immunoglobulin superfamily*" OR "importin*" OR "Inhibin*" OR "inositol monophosphatase" OR "somatomedin*" OR "Insulin-like growth factor*" OR "Insulinlike growth factor*" OR "Insulysin" OR "Integrin*" OR "inter alpha trypsin inhibitor*" OR "intercellular adhesion molecule 1" OR "interleukin*" OR "Interphotoreceptor matrix proteoglycan" OR "Interstitial collagenase " OR "irisin" OR "Isocitrate dehydrogenase" OR "isthmin" OR "Kallikrein" OR "Kallmann syndrome 1 protein" OR "Kazal-type serine peptidase inhibitor domain" OR "Kelch repeat and BTB domain-containing protein" OR "keratin" OR "keratinocyte growth factor" OR "KIAA0090 protein" OR "KIAA0564 protein" OR "KIAA0922 protein" OR "KIAA1199 protein" OR "KIAA1233 protein" OR "chordin-like protein" OR "kielin-like protein" OR "kinase insert domain receptor" OR "kinectin" OR "kit ligand" OR "kynurenic acid" OR "kynurenine" OR "kinurenine" OR "Lactadherin " OR "lactotransferrin" OR "ladinin*" OR "laminin*" OR "Latent transforming growth factor beta binding protein" OR "Legumain " OR "leptin" OR "leucine rich repeat containing*" OR "leucine-rich alpha-2-glycoprotein 1" OR "leukemia inhibitory factor" OR "leukaemia inhibitory factor" OR "leukocyte elastase inhibitor" OR "leukocyte immunoglobulin like receptor*" OR "lipase" OR "lipocalin*" OR "liver carboxylesterase*" OR "L-lactate" OR "Lactate" OR "Lactic Acid" OR "low density lipoprotein receptor" OR "lumican" OR "lutropin subunit beta" OR "lymphocyte antigen 6 complex" OR "lymphotoxin*" OR "lysine demethylase*" OR "lysosomal protective protein" OR "lysozyme" OR "lysyl oxidase*" OR "macrophage colony-stimulating factor*" OR "Macrophage migration inhibitory factor*" OR "major histocompatibility complex*" OR "Malignant fibrous histiocytoma amplified sequence*" OR "MAM domain-containing protein*" OR "mammalian ependymin-related protein*" OR "mannan binding lectin serine*" OR "Matrilin*" OR "matrilysin" OR "Matrix Gla protein" OR "matrix metallopeptidase*" OR "matrix-remodelling associated*" OR "mesoderm specific transcript" OR "Metalloproteinase inhibitor*" OR "meteorin-like*" OR "glial cell differentiation regulator-like*" OR "MHC class I polypeptide-related sequence*" OR "microfibril-associated glycoprotein*" OR "Microfibrillar-associated protein*" OR "Microfibrillar-associated protein*" OR "moesin" OR "monocyte chemoattractant protein*" OR "monocyte chemotactic protein*" OR "MT-RNR2-like*" OR "Mucin*" OR "Musculin*" OR "multimerin*" OR "Multiple coagulation factor deficiency protein*" OR "Multisynthetase complex auxiliary component*" OR "MVAL564" OR "myonectin" OR "myosin light chain*" OR "myosin regulatory light chain*" OR "myostatin" OR "dimethylaminohydrolase*" OR "N-acetyl-D-glucosamine kinase" OR "N-acetylglucosamine-1-phosphotransferase" OR "NADH dehydrogenase*" OR "NADPH dehydrogenase*" OR "natural killer cell granule protein" OR "nectin cell adhesion molecule*" OR "Nel-like*" OR "Netrin*" OR "neural cell adhesion molecule*" OR "neuregulin*" OR "neurexophilin*" OR "Neuroendocrine protein*" OR "Neuronal growth regulator*" OR "neuronal pentraxin*" OR "neuropilin*" OR "Neurotrimin" OR "neurotrophin*" OR "motopsin" OR "brain-specific serine protease*" OR "leydin" OR "Neurotrypsin" OR "neutrophil collagenase*" OR "NFAT activating protein with ITAM motif*" OR "nicotinamide phosphoribosyl transferase" OR "enactin" OR "Nidogen*" OR "Niemann-Pick disease" OR "NKG2D ligand*" OR "Non-specific cross reacting antigen" OR "notch 4" OR "NS5ATP13TP2 protein" OR "Nucleobindin*" OR "olfactomedin*" OR "oncostatin*" OR "osteoglycin" OR "osteomodulin" OR "Out at first protein homolog " OR "Oxysterol binding protein*" OR "Palmitoyl-protein thioesterase*" OR "Pappalysin*" OR "Paraoxonase*" OR "pentraxin*" OR "peptidase domain containing associated with muscle regeneration 1" OR "Peptidyl-glycine alpha-amidating monooxygenase" OR "peptidyl-prolyl cis-trans isomerase*" OR "perforin*" OR "Perilipin*" OR "periostin" OR "peripherin" OR "peroxidasin" OR "peroxiredoxin*" OR "phosphatidylethanolamine-binding protein*" OR "phosphodiesterase 7A" OR "phosphofructokinase" OR "phosphoinositide-3-kinase interacting protein 1" OR "Phospholipase A2" OR "phospholipid transfer protein" OR "Pigment epithelium-derived factor*" OR "pituitary tumor-transforming 1 interacting protein" OR "PKCl-1-related HIT protein" OR "placental growth factor" OR "Plasma alpha-L-fucosidase " OR "Plasma glutamate carboxypeptidase" OR "Plasma glutathione peroxidase*" OR "plasma protease C1 inhibitor" OR "plasminogen*" OR "platelet derived growth factor*" OR "pleiotrophin" OR "pM5 protein" OR "Poliovirus receptor " OR "Poly(ADP-ribose) glycohydrolase ARH3" OR "poly(rC)-binding protein 1" OR "polymerase I and transcript release factor" OR "polypeptide N-acetylgalactosaminyltransferase*" OR "prefoldin" OR "Pregnancy-associated plasma protein A" OR "prenylcysteine oxidase 1 like" OR "proactivator polypeptide" OR "Procollagen C-endopeptidase enhancer 1 " OR "procollagen-lysine,2-oxoglutarate 5-dioxygenase 1" OR "Pro-epidermal growth factor" OR "epidermal growth factor" OR "profilin-2" OR "proheparin-binding EGF-like growth factor" OR "Proline arginine-rich end leucine-rich repeat protein ; prolargin; 55 kDa leucine-rich repeat protein of articular cartilage" OR "Proline rich 3" OR "Proline-rich protein" OR "salivary protein" OR "parotid middle band protein" OR "prolow-density lipoprotein receptor-related protein*" OR "sphingolipid activator protein*" OR "Proprotein convertase subtilisin*" OR "Prosaposin" OR "prostaglandin I2 receptor" OR "prostacyclin receptor" OR "prostaglandin-endoperoxide synthase 2" OR "prostaglandin-H2 D-isomerase" OR "Prostate specific antigen*" OR "Prostatic acid phosphatase" OR "Serine protease" OR "Protease-associated domain-containing protein" OR "proteasome subunit beta*" OR "Protein ARMET" OR "protein disulfide-isomerase*" OR "protein FAM3C" OR "Protein HEG homolog 1 " OR "Protein jagged-1 " OR "Protein KIAA1199 " OR "protein S100*" OR "protein SEC13 homolog" OR "protein tyrosine phosphatase" OR "Protein-lysine 6-oxidase" OR "proteoglycan*" OR "Prothyroliberin" OR "puromycin-sensitive aminopeptidase" OR "Putative nucleoside diphosphate kinase" OR "Putative phospholipase B-like 2" OR "putative quinone oxidoreductase" OR "Putative secreted protein ZSIG11" OR "Pyridoxal kinase" OR "quinolinic acid" OR "ras suppressor protein 1" OR "RBP4 gene product" OR "receptor accessory protein 2" OR "Reelin isoform a" OR "regulated on activation, normal T cell expressed and secreted" OR "RELT tumor necrosis factor receptor" OR "reticulocalbin*" OR "Retinal dehydrogenase*" OR "Retinoic acid receptor responder*" OR "RGM domain family*" OR "Ribonuclease*" OR "ring finger protein*" OR "S100 calcium binding protein*" OR "Salivary acidic proline-rich phosphoprotein*" OR "SEC16 homolog B" OR "endoplasmic reticulum export factor" OR "secernin*" OR "Secretad protein of unknown function" OR "secreted and transmembrane*" OR "secreted frizzled related protein*" OR "Secreted apoptosis-related protein*" OR "secreted protein acid and rich in cystein" OR "secretin" OR "Secretogranin*" OR "secretory leukocyte peptidase inhibitor" OR "Selenoprotein*" OR "sema domain 3F" OR "Semaphorin*" OR "serglycin" OR "Serine proteinase inhibitor" OR "cysteine proteinase inhibitor" OR "Serine carboxypeptidase*" OR "serine peptidase inhibitor*" OR "Serine protease*" OR "threonine-protein phosphatase" OR "serpin H1" OR "serpin peptidase inhibitor*" OR "antitrypsin" OR "alpha-1 antiproteinase" OR "α1 antiproteinase" OR "nexin" OR "alpha-2 antiplasmin" OR "α2 antiplasmin" OR "serum albumin" OR "serum amyloid A*" OR "tumor protein p53 inducible protein*" OR "sestrin" OR "SET07 protein" OR "S-formylglutathione hydrolase" OR "SH3 domain-binding glutamic acid-rich-like protein" OR "sialate O-acetylesterase" OR "sialic acid binding Ig like lectin*" OR "EGF-like domain-containing protein 3 " OR "Similar to common salivary protein 1" OR "single-stranded DNA-binding protein" OR "Slit homolog*" OR "Small EDRK-rich factor 2" OR "Smhs2 homolog" OR "Soluble calcium-activated nucleotidase*" OR "solute carrier family*" OR "somatotropin" OR "sorcin" OR "SPARC" OR "testican" OR "osteonectin*" OR "kazal-like domains proteoglycan" OR "spermatogenesis cell proliferation related protein" OR "Spermatogenesis associated*" OR "sphingomyelin phosphodiesterase*" OR "spliceosome RNA helicase DDX39B" OR "spondin-2" OR "ST3 beta-galactoside alpha-2,3-sialyltransferase*" OR "ST6 beta-galactoside alpha-2,6-sialyltransferase*" OR "ST6 N-acetylgalactosaminide alpha-2,6-sialyltransferase*" OR "ST8 alpha-N-acetyl-neuraminide alpha-2,8-sialyltransferase*" OR "stanniocalcin*" OR "Statherin" OR "stathmin" OR "stress-70 protein" OR "Stromal cell-derived factor*" OR "Sulfatase 1" OR "Sulfatase modifying factor*" OR "sulfhydryl oxidase*" OR "superoxide dismutase" OR "sushi repeat containing protein*" OR "Sushi-repeat protein*" OR "SYAP1 protein" OR "syndecan*" OR "synuclein, alpha" OR "target of Nesh-SH3" OR "T-complex protein 1 subunit theta" OR "TEK tyrosine kinase, endothelial" OR "Tenascin*" OR "cytotactin" OR "Hexabrachion*" OR "Testican-1 " OR "tetranectin" OR "Tetratricopeptide repeat domain*" OR "thioredoxin domain-containing protein 5" OR "thioredoxin-dependent peroxide reductase" OR "thrombomodulin" OR "thrombospondin*" OR "Thy-1 cell surface antigen" OR "Thyroglobulin" OR "thyroid peroxidase" OR "thyrotropin*" OR "TIMP metallopeptidase inhibitor*" OR "tissue factor pathway inhibitor*" OR "tissue inhibitor of metalloproteinase*" OR "TNF alpha induced protein*" OR "toll like receptor*" OR "torsin family 3*" OR "tranlationally-controlled tumor protein" OR "Transferrin*" OR "Transforming growth factor beta* " OR "Transforming growth factor β* " OR "transgelin*" OR "transmembrane protein*" OR "triggering receptor expressed on myeloid cells*" OR "tripartite motif-containing protein*" OR "Tripeptidyl-peptidase*" OR "trombopoietin*" OR "tropomyosin*" OR "troponin I" OR "troponin T" OR "tubulin beta*" OR "tubulin β*" OR "tumor necrosis factor*" OR "PDGFA associated protein*" OR "Tumor suppressor candidate*" OR "Twisted gastrulation*" OR "TYRO protein tyrosine kinase binding protein*" OR "tyrosinase-related protein*" OR "ubiquitin*" OR "UMP-CMP kinase" OR "UPF0556 protein C19orf10" OR "Urocortin" OR "vanin*" OR "Vascular cell adhesion protein*" OR "vascular endothelial growth factor*" OR "vasculotropin*" OR "vasoactive intestinal peptide*" OR "vasorin*" OR "vimentin*" OR "visfatin*" OR "vitamin K-dependent protein S" OR "vitelline membrane outer layer 1 homolog" OR "Vitrin*" OR "vitronectin*" OR "Von Willebrand factor*" OR "V-set and immunoglobulin domain containing*" OR "WD repeat domain*" OR "WD repeat-containing protein*" OR "wingless-type MMTV integration site family*" OR "Wnt family member*" OR "Wnt inhibitory factor*" OR "WNT1 inducible signaling pathway protein*" OR "wnt-1 signaling pathway protein*" OR "XTP3-transactivated protein B" OR "Xylosyltransferase 1" OR "Zinc finger protein*" OR "ABO" OR "ADAM8" OR "ADAMTS9" OR "ADAMTS1" OR "ADAMTS15" OR "ADAMTS2" OR "ADAMTS4" OR "ADAMTS5 " OR "ADAMTS7" OR "ADAMTS8" OR "ADAMTSL3" OR "ADAMTSL1" OR "ADCYAP1R1" OR "AMICA1" OR "ADIPOQ" OR "ADM" OR "AEBP1" OR "AGRN" OR "A2M" OR "ANGPTL2" OR "ANGPTL4" OR "ANGPTL7" OR "ACE" OR "ASB14" OR "APLN" OR "APOLD1" OR "ARSI" OR "ASPN" OR "BAIBA" OR "MANBA" OR "BGN" OR "BMP1" OR "BMP2" OR "BMP5" OR "BDNF" OR "BTN3A1" OR "BTN3A3" OR "CDH24" OR "CDH5" OR "CALCR" OR "CES3" OR "CPXM1" OR "CEACAM1" OR "CLCF1" OR "COMP" OR "CTSB" OR "CTSC" OR "CTSD" OR "CTSH" OR "CTSK" OR "CTSL" OR "CTSL1" OR "CTSL2" OR "CTSS" OR "CTSZ" OR "CCL1" OR "CCL15" OR "CCL17" OR "CCL18" OR "CCL2" OR "CCL21" OR "CCL23" OR "CCL24" OR "CCL26" OR "CCL3" OR "CCL4" OR "CCL5" OR "CCL8" OR "CCL21" OR "CD163L1" OR "CD200" OR "CD300LG" OR "CX3CL1" OR "CXCL1" OR "CXCL10" OR "CXCL12" OR "CXCL13" OR "CXCL2" OR "CXCL3" OR "CXCL5" OR "CXCL8" OR "CXCL9" OR "CHAD" OR "CSGALNACT1" OR "CHSY1" OR "CHGB" OR "C1orf127" OR "CNTFR" OR "F2R" OR "CCDC80" OR "COL1A1" OR "CTHRC1" OR "COL1A1" OR "COL1A2" OR "COL3A1" OR "COL4A1" OR "COL4A2" OR "COL5A1" OR "COL5A2" OR "COL6A6" OR "COL14A1" OR "COL15A1" OR "CSF1" OR "CSF1R" OR "CSF2RB" OR "CSF3" OR "CSF3R" OR "C1QTNF" OR "Complement C3" OR "C8G" OR "CFP" OR "CTGF" OR "CNTNAP3B" OR "CRHR2" OR "CRP" OR "CLEC11A" OR "CLEC2D" OR "CYR61" OR "CRISPLD2" OR "CYR61" OR "CYP2E1" OR "POR" OR "DHRS4L2" OR "DLL1" OR "DFNA5" OR "DNAJB9" OR "DUSP6" OR "DYRK2" OR "ELN" OR "EMILIN3" OR "ENAM" OR "EDN1" OR "EPHA2" OR "EPHA3" OR "EPHB1" OR "ERBB3" OR "ECM1" OR "ECM2" OR "F2RL3" OR "FAM20C" OR "FAM57A" OR "FAM57B" OR "FAT1" OR "FCGR2A" OR "FCGR2B" OR "FCGR3A" OR "FCGR3B" OR "FBN1" OR "FBN2" OR "FBN3" OR "FBN3" OR "FGF10" OR "FGF18" OR "FGF21" OR "FGF17" OR "FGF4" OR "FGF2" OR "FGF6" OR "FGF9" OR "FGFR3" OR "FNDC5" OR "FCN1" OR "FCN3" OR "FKBP7" OR "FKBP3" OR "FLT1" OR "FSTL1" OR "FJX1" OR "FKN" OR "FRZB" OR "FUT1" OR "GPR162" OR "GLA" OR "GABRE" OR "GSN" OR "GDNF" OR "GRM2" OR "GRIN2C" OR "GFPT2" OR "QPCT" OR "CGA" OR "GCSF" OR "GNLY" OR "GREM2" OR "GREM1" OR "GDF-15" OR "GDF-8" OR "GBP1" OR "HSPH1" OR "HBA2" OR "HSPG2" OR "HEPH" OR "H6PD" OR "HAPLN3" OR "HAS3" OR "HSD17B13" OR "LOC100505657" OR "IFNG" OR "IL-16" OR "IL-22" OR "IL-28A" OR "IL-29" OR "IL-3" OR "IL-31" OR "ISLR2" OR "IGSF10" OR "INHBB" OR "INHBE" OR "IGF1" OR "IGF2" OR "IGFBP2" OR "IGFBP3" OR "IGFBP4" OR "IGFBP5" OR "IGFBP6" OR "IGFBP7" OR "IGFBP1" OR "ITGA5" OR "ITGB7" OR "ITIH3" OR "ICAM1" OR "IFI30" OR "IL1RN" OR "IL1R1" OR "IL1B" OR "IL10RA" OR "IL-13" OR "IL-15" OR "IL-17A" OR "IL-17D" OR "IL-2" OR "IL2RB" OR "IL2RB" OR "IL-33" OR "IL-4" OR "IL4R" OR "IL-6" OR "IL6R" OR "IL7R" OR "IL-8" OR "IL-1B" OR "IL-10" OR "IL-12" OR "IL-13" OR "IL-28A" OR "IL-29" OR "IL-3" OR "IL-31" OR "IL-7" OR "ISM1" OR "KAZALD1" OR "KIAA0922" OR "KCP" OR "KDR" OR "LTF" OR "LAD1" OR "LAMA3" OR "LAMA2" OR "LAMA4" OR "LAMA5" OR "LAMB3" OR "LAMB1" OR "LAMB2" OR "LAMC1" OR "LAMA1" OR "LAMC2" OR "LAMC3" OR "LRRC17" OR "LRRC32" OR "LRRC3B" OR "LRG1" OR "LIF" OR "LILRA6" OR "LILRB3" OR "LIPG" OR "LCN10" OR "LCN6" OR "LPL" OR "LDLR" OR "LUM" OR "LY6G5B" OR "KDM4A" OR "LYZ" OR "LOX" OR "LOXL2" OR "LOXL3" OR "HLA-G" OR "MASP2" OR "MMP14" OR "MMP19" OR "MMP25" OR "MMP28" OR "MMP1" OR "MMP2" OR "MXRA5" OR "MEST" OR "METRNL" OR "MCP-1" OR "MCP-4" OR "MTRNR2L4" OR "MTRNR2L6" OR "MUC6" OR "MMRN1" OR "MSTN" OR "NKG7" OR "PVRL2" OR "NRG2" OR "NXPH3" OR "NPTX2" OR "NRP2" OR "NFAM1" OR "NAMPT" OR "NID1" OR "NID2" OR "NOTCH4" OR "OLFM1" OR "OLFML1" OR "OLFML2B" OR "OGN" OR "OMD" OR "PTX3" OR "PAMR1" OR "PRF1" OR "POSTN" OR "PXDN" OR "PDE7A" OR "PFKP" OR "PIK3IP1" OR "PLA2G15" OR "PTTG1IP" OR "PGF" OR "PLG" OR "PLAU" OR "PLAUR" OR "PDGFB" OR "PDGFA" OR "PDGFB" OR "PTN" OR "GALNT15" OR "PCYOX1L" OR "PLOD2" OR "PTGIR" OR "PTGS2" OR "PRSS42" OR "PTPRN2" OR "PRG4" OR "REEP2" OR "RANTES" OR "RELT" OR "RARRES1" OR "RARRES2" OR "RNF24" OR "RNF43" OR "S100A8" OR "S100A9" OR "SEC16B" OR "SECTM1" OR "SFRP4" OR "SFRP5" OR "SFRP1" OR "SFRP2" OR "SFRP2" OR "SPARC" OR "SCT" OR "SLPI" OR "SEMA3F" OR "SEMA3G" OR "SEMA4C" OR "SEMA4G" OR "SEMA5A" OR "SEMA6D" OR "SEMA7A" OR "SRGN" OR "SPINK5" OR "SERPINH1" OR "SERPINA1" OR "SERPINA3" OR "SERPINE1" OR "SERPINF2" OR "SAA1" OR "SAA2" OR "SIGLEC1" OR "SLC39A14" OR "SLC45A4" OR "SLC6A8" OR "SMPDL3A" OR "ST3GAL1" OR "ST6GAL1" OR "ST6GALNAC1" OR "ST8SIA2" OR "STC1" OR "STC2" OR "SDF-1" OR "SRPX" OR "SRPX2" OR "SDC4" OR "SNCA" OR "TEK" OR "TNC" OR "TNC" OR "THBD" OR "THBS1" OR "THBS2" OR "THBS3" OR "THBS4" OR "THY1" OR "TPO" OR "TFPI2" OR "TIMP1" OR "TIMP2" OR "TIMP3" OR "TNFAIP6" OR "TLR9" OR "TOR3A" OR "TGFB1" OR "TGFB2" OR "TGFB3" OR "TMEM119" OR "TREM1" OR "TNFSF10" OR "TNF " OR "TNFRSF10C" OR "TNFRSF12A" OR "TNFRSF8" OR "TNFRSF1B" OR "TNFRSF25" OR "TNF9" OR "TYROBP" OR "TYRP1" OR "VNN2" OR "VEGFA" OR "VEGFC" OR "VIPR1" OR "VMO1" OR "VWA1" OR "VSIG10" OR "WDR81" OR "WNT9A" OR "WNT5A" OR "WISP1" OR "ADAM-8" OR "ADAMTS-9" OR "ADAMTS-1" OR "ADAMTS-15" OR "ADAMTS-2" OR "ADAMTS-4" OR "ADAMTS-5 " OR "ADAMTS-7" OR "ADAMTS-8" OR "ADAMTS-L3" OR "ADAMTS-L1" OR "AMICA-1" OR "AEBP-1" OR "ANGPT-L2" OR "ANGPT-L4" OR "ANGPT-L7" OR "ACE-inhibitor" OR "ASB-14" OR "APOLD-1" OR "BMP-1" OR "BMP-2" OR "BMP-5" OR "BTN3-A1" OR "BTN3-A3" OR "CDH-24" OR "CDH-5" OR "CES-3" OR "CPXM-1" OR "CEACAM-1" OR "CLCF-1" OR "CTSL-1" OR "CTSL-2" OR "CCL-1" OR "CCL-15" OR "CCL-17" OR "CCL-18" OR "CCL-2" OR "CCL-21" OR "CCL-23" OR "CCL-24" OR "CCL-26" OR "CCL-3" OR "CCL-4" OR "CCL-5" OR "CCL-8" OR "CCL-21" OR "CD163-L1" OR "CX3CL-1" OR "CXCL-1" OR "CXCL-10" OR "CXCL-12" OR "CXCL-13" OR "CXCL-2" OR "CXCL-3" OR "CXCL-5" OR "CXCL-8" OR "CXCL-9" OR "COL1A-1" OR "CTHRC-1" OR "COL1A-2" OR "COL3A-1" OR "COL4A-1" OR "COL4A-2" OR "COL5A-1" OR "COL5A-2" OR "COL6A-6" OR "COL14A-1" OR "COL15A-1" OR "CSF-1" OR "CSF-1R" OR "CSF-2RB" OR "CSF-3" OR "CSF-3R" OR "C1Q-TNF" OR "CNTNAP-3B" OR "CRHR-2" OR "CLEC-11A" OR "CLEC-2D" OR "CYR-61" OR "CRISPLD-2" OR "CYR-61" OR "CYP-2E1" OR "POR" OR "DHRS-4L2" OR "DLL-1" OR "DFNA-5" OR "DNAJB-9" OR "DUSP-6" OR "DYRK-2" OR "EMILIN-3" OR "ENAM" OR "EDN-1" OR "EPHA-2" OR "EPHA-3" OR "EPHB-1" OR "ERBB-3" OR "ECM-1" OR "ECM-2" OR "F2RL-3" OR "FAM-20C" OR "FAM-57A" OR "FAM-57B" OR "FAT-1" OR "FCGR-2A" OR "FCGR-2B" OR "FCGR-3A" OR "FCGR-3B" OR "FBN-1" OR "FBN-2" OR "FBN-3" OR "FGF-10" OR "FGF-18" OR "FGF-21" OR "FGF-4" OR "FGF-2" OR "FGF-6" OR "FGF-9" OR "FGFR-3" OR "FNDC-5" OR "FCN-1" OR "FCN-3" OR "FKBP-7" OR "FKBP-3" OR "FLT-1" OR "FSTL-1" OR "FJX-1" OR "FUT-1" OR "GPR-162" OR "GRM-2" OR "GRIN-2C" OR "GFPT-2" OR "GREM-2" OR "GREM-1" OR "GDF15" OR "GDF8" OR "GBP-1" OR "HSPH-1" OR "HBA-2" OR "HSPG-2" OR "HAPLN-3" OR "HAS-3" OR "HSD-17B13" OR "LOC100505657" OR "IFNG" OR "IL16" OR "IL22" OR "IL28A" OR "IL29" OR "IL3" OR "IL31" OR "ISLR-2" OR "IGSF-10" OR "IGF-1" OR "IGF-2" OR "IGFBP-2" OR "IGFBP-3" OR "IGFBP-4" OR "IGFBP-5" OR "IGFBP-6" OR "IGFBP-7" OR "IGFBP-1" OR "ITGA-5" OR "ITGB-7" OR "ITIH-3" OR "ICAM-1" OR "IFI-30" OR "IL-1RN" OR "IL-1R1" OR "IL-1B" OR "IL-10RA" OR "IL13" OR "IL15" OR "IL17A" OR "IL17D" OR "IL2" OR "IL-2RB" OR "IL33" OR "IL4" OR "IL-4R" OR "IL6" OR "IL-6R" OR "IL-7R" OR "IL8" OR "IL1B" OR "IL10" OR "IL12" OR "IL28A" OR "IL29" OR "IL3" OR "IL31" OR "IL7" OR "ISM-1" OR "KAZALD-1" OR "LAD-1" OR "LAMA-3" OR "LAMA-2" OR "LAMA-4" OR "LAMA-5" OR "LAMB-3" OR "LAMB-1" OR "LAMB-2" OR "LAMC-1" OR "LAMA-1" OR "LAMC-2" OR "LAMC-3" OR "LRRC-17" OR "LRRC-32" OR "LRRC-3B" OR "LRG-1" OR "LILRA-6" OR "LILRB-3" OR "LCN-10" OR "LCN-6" OR "LY6G-5B" OR "KDM-4A" OR "LOXL-2" OR "LOXL-3" OR "HLAG" OR "MASP-2" OR "MMP-14" OR "MMP-19" OR "MMP-25" OR "MMP-28" OR "MMP-1" OR "MMP-2" OR "MXRA-5" OR "MCP1" OR "MCP4" OR "MTRNR-2L4" OR "MTRNR-2L6" OR "MUC-6" OR "MMRN-1" OR "NKG-7" OR "PVRL-2" OR "NRG-2" OR "NXPH-3" OR "NPTX-2" OR "NRP-2" OR "NFAM-1" OR "NAMPT" OR "NID-1" OR "NID-2" OR "NOTCH-4" OR "OLFM-1" OR "OLFM-L1" OR "OLFM-L2B" OR "PTX-3" OR "PAMR-1" OR "PRF-1" OR "PDE-7A" OR "PIK3IP-1" OR "PLA2G-15" OR "GALNT-15" OR "PCYOX-1L" OR "PLOD-2" OR "PTGS-2" OR "PRSS-42" OR "PTPRN-2" OR "PRG-4" OR "REEP-2" OR "RARRES-1" OR "RARRES-2" OR "RNF-24" OR "RNF-43" OR "S100-A8" OR "S100-A9" OR "SEC-16B" OR "SECTM-1" OR "SFRP-4" OR "SFRP-5" OR "SFRP-1" OR "SFRP-2" OR "SFRP-2" OR "SEMA-3F" OR "SEMA-3G" OR "SEMA-4C" OR "SEMA-4G" OR "SEMA-5A" OR "SEMA-6D" OR "SEMA-7A" OR "SPINK-5" OR "SERPIN-H1" OR "SERPIN-A1" OR "SERPIN-A3" OR "SERPIN-E1" OR "SERPIN-F2" OR "SAA-1" OR "SAA-2" OR "SIGLEC-1" OR "SLC39A-14" OR "SLC45A-4" OR "SLC6A-8" OR "SMPDL-3A" OR "ST3GAL-1" OR "ST6GAL-1" OR "ST6GALNAC-1" OR "ST8SIA-2" OR "STC-1" OR "STC-2" OR "SDF1" OR "SRPX-2" OR "SDC-4" OR "THBS-1" OR "THBS-2" OR "THBS-3" OR "THBS-4" OR "THY-1" OR "TFPI-2" OR "TIMP-1" OR "TIMP-2" OR "TIMP-3" OR "TNFAIP-6" OR "TLR-9" OR "TOR-3A" OR "TGF-B1" OR "TGF-B2" OR "TGF-B3" OR "TMEM-119" OR "TREM-1" OR "TNFSF-10" OR "TNF-α" OR "TNFα" OR "TNFRSF-10C" OR "TNFRSF-12A" OR "TNFRSF-8" OR "TNFRSF-1B" OR "TNFRSF-25" OR "TNF-9" OR "TYRP-1" OR "VNN-2" OR "VEGF-A" OR "VEGF-C" OR "VEGF" OR "VIPR-1" OR "VMO-1" OR "VWA-1" OR "VSIG-10" OR "WDR-81" OR "WNT-9A" OR "WNT-5A" OR "WISP-1")

Index terms per database:

1. PubMed:

"Aged"[Mesh] OR "Aged, 80 and over"[Mesh] OR "Aging"[Mesh] OR "Middle Aged"[Mesh]

AND

"Exercise"[Mesh] OR "Exercise Therapy"[Mesh] OR "Sports"[Mesh] OR "Exercise Test"[Mesh]

AND

"Mental Processes"[Mesh] OR "Attention"[Mesh]

AND

"Brain-Derived Neurotrophic Factor"[Mesh] OR "Insulin-Like Growth Factor I"[Mesh] OR "Cytokines"[Mesh] OR "Adiponectin"[Mesh] OR "Myostatin"[Mesh] OR "Apelin"[Mesh] OR "Fibroblast Growth Factor 2"[Mesh] OR "Osteonectin"[Mesh] OR "Lactic Acid"[Mesh] OR "Leukemia Inhibitory Factor"[Mesh] OR "Chemokine CCL2"[Mesh] OR "Kynurenine"[Mesh] OR "TGF-beta Superfamily Proteins"[Mesh] OR "Vascular Endothelial Growth Factors"[Mesh] OR "Metrnl protein, human" [Supplementary Concept] OR "Chemokine CX3CL1"[Mesh] OR "Chemokine CXCL12"[Mesh] OR "Decorin"[Mesh] OR "Chemokine CCL5"[Mesh]

1. Embase

'middle aged'/exp OR 'aged'/exp OR 'aging'/exp

AND

'exercise'/exp OR 'sport'/exp OR 'physical activity'/exp

AND

'cognition'/exp

AND

'brain derived neurotrophic factor'/exp OR 'somatomedin C'/exp OR 'cytokine'/exp OR 'myostatin'/exp OR 'apelin'/exp OR 'fibroblast growth factor'/exp OR 'osteonectin'/exp OR 'lactic acid'/exp OR 'leukemia inhibitory factor'/exp OR 'kynurenine'/exp OR 'transforming growth factor beta'/exp OR 'vasculotropin'/exp OR 'decorin'/exp OR 'cathepsin B'/exp OR 'irisin'/exp OR 'monocyte chemotactic protein'/exp OR 'angiopoietin related protein 4'/exp OR 'bone morphogenetic protein'/exp OR 'stromal cell derived factor 1'/exp

1. PsycInfo

DE "Aging" OR DE "Geriatrics" OR DE "Older Adulthood"

AND

DE "Exercise" OR DE "Sports"

AND

DE "Cognition" OR DE "Cognitive Processes" OR DE "Cognition" OR DE "Cognitions" OR DE "Cognitive Ability" OR DE "Cognitive Flexibility" OR DE "Cognitive Processing Speed" OR DE "Concentration" OR DE "Decision Making" OR DE "Executive Function" OR DE "Problem Solving" OR DE "Cognitive Assessment" OR DE "Cognitive Measures" OR DE "Executive Functioning Measures" OR DE "Memory and Learning Measures" OR DE "Neuropsychological Assessment" OR DE "Mini Mental State Examination" OR DE "Task Switching" OR DE "Wisconsin Card Sorting Test" OR DE "Memory" OR DE "Learning" OR DE "Attention" OR DE "Neurocognition"

AND

DE "Cytokines" OR DE "Interleukins" OR DE "Tumor Necrosis Factor" OR DE "Brain Derived Neurotrophic Factor" OR DE "Insulin-like Growth Factor" OR DE "Lactic Acid"

1. SportDiscuss

DE "AGING" OR DE "OLDER people"

AND

DE "EXERCISE" OR DE "SPORTS"

AND

DE "COGNITION" OR DE "EFFECT of exercise on cognition" OR DE "MEMORY" OR DE "ATTENTION"

AND

(DE "SOMATOMEDIN") OR (DE "ADIPONECTIN")

1. CINAHL

(MH "Aged+") OR (MM "Middle Age")

AND

(MH "Physical Therapy+") OR (MH "Exercise+") OR (MH "Sports+") OR (MH "Exercise Test+")

AND

(MH "Mental Processes+") OR (MH "Attention+")

AND

(MM "Brain-Derived Neurotrophic Factor") OR (MH "Cytokines+") OR (MM "Somatomedins") OR (MM "Tumor Necrosis Factor") OR (MM "Vascular Endothelial Growth Factors") OR (MM "Myostatin") OR (MM "Adiponectin") OR (MH "Fibroblast Growth Factors+") OR (MM "Lactic Acid") OR (MM "Transforming Growth Factor beta")

1. Cochrane Library (accessed to search CT.gov and ICTRP)

[mh "Aged"] OR [mh "Aged, 80 and over"] OR [mh "Aging"] OR [mh "Middle Aged"]

AND

[mh "Exercise"] OR [mh "Exercise Therapy"] OR [mh "Sports"] OR [mh "Exercise Test"]

AND

[mh "Mental Processes"] OR [mh "Attention"]

AND

[mh "Brain-Derived Neurotrophic Factor"] OR [mh "Insulin-Like Growth Factor I"] OR [mh "Cytokines"] OR [mh "Adiponectin"] OR [mh "Myostatin"] OR [mh "Apelin"] OR [mh "Fibroblast Growth Factor 2"] OR [mh "Osteonectin"] OR [mh "Lactic Acid"] OR [mh "Leukemia Inhibitory Factor"] OR [mh "Chemokine CCL2"] OR [mh "Kynurenine"] OR [mh "TGF-beta Superfamily Proteins"] OR [mh "Vascular Endothelial Growth Factors"] OR [mh "Chemokine CX3CL1"] OR [mh "Chemokine CXCL12"] OR [mh "Decorin"] OR [mh "Chemokine CCL5"]
